# Evaluating Youtube as A Source of Patient Information on Dupuytren’s Disease

**Published:** 2017-09

**Authors:** Matt Jones, Akira Wiberg

**Affiliations:** 1 Orthopaedic Department, Princess Elizabeth Orthopaedic Centre, Royal Devon and Exeter Hospital, Exeter, UK;; 2 Botnar Research Centre, NDORMS, University of Oxford, Nuffield Orthopaedic Centre, Oxford, UK

**Keywords:** Dupuytren’s disease, Youtube, Patient information, Collagenase

**DEAR EDITOR**

Dupuytren’s disease (DD) is a benign, fibroproliferative disorder of the hand resulting in contracture of the digits. Traditionally, surgical treatment (fasciectomy or dermofasciectomy) has been the gold standard. Needle aponeurotomy and collagenase injections are newer alternatives with potentially higher recurrence rates and lower efficacy in severe cases.^1^^,^^2^ There is no consensus on a single superior treatment for DD.

Youtube is a free, publically accessible website where users upload and view videos on various topics including healthcare. It is an unregulated information source, making it difficult to verify the credibility of its content.^3^ With potentially unreliable information being accessed by patients, it is important to assess what is being viewed. We aimed to analyse the content of videos on Youtube concerning DD. 

YouTube was searched using the terms: “Dupuytren’s contracture”, “Dupuytren’s disease” and “Dupuytren’s treatment”. Results were sorted by relevance, the first 40 videos from each search were included and duplicate videos or videos without dialogue were excluded (n=55). Two medical professionals independently assessed the source, content and educational quality of 55 videos. The source of a video was determined by identifying those who featured in the video or those who had uploaded the content, through the information available to the viewer. The source of each video was divided into medical professional, news/TV, patient/public and non-profit organisation.

Content was assessed for the mention of general background information on DD, surgical treatment options, needle aponeurotomy, collagenase injections and other treatment alternatives. It was noted if a video only had a single focus or was an advertisement. Educational quality was assessed and divided into the following categories; useful to patients, useful only to medical professionals, not useful or misleading. For a video to be deemed “useful to patients” it had to be scientifically accurate and in keeping with what is widely accepted in literature about DD. 

Additionally, it had to avoid the use of medical jargon and assume no prior medical knowledge; otherwise, it would be considered “useful only to medical professionals”. Videos were “not useful” if the information was solely anecdotal, with no emphasis on education about the disease or treatment options. “Misleading” videos either failed deliver accurate disease information or promoted treatment with no evidence base. 

The majority of videos were uploaded by medical professionals (34), with the remainder by News/TV (10), patient/public (9) or non-profit organisations (2). Seventy percent of videos that were “useful to patients” were produced by medical professionals. Seventy-six percent of videos focused only on one treatment modality, of which 46% were on collagenase injections. These videos had the least number of mean views and more than half were advertisements.

Those video s “only useful to medical professionals” were all produced by medical professionals; they provided less general disease information (43%) and focused on the technicalities of surgical treatments (71%). They also received the highest number of mean views, although had the fewest number of mean “likes”. The videos deemed “not useful” only discussed treatment options, with 80% having a focus on only one treatment modality, 50% of which focused on collagenase injections (Table 1). 

**Table 1:** Analysis of videos on the topic of Dupuytren’s disease in relation to education quality.

**Video characteristics**	**Educational Quality**				**Total**
	**Useful to patients**	**Useful only to medical professionals**	**Not useful**	**Misleading**	
Videos [no. (%)]	30	7	10	8	55
Source					
Medical professional	21 (70)	7 (100)	5 (50)	1 (13)	34
News/ TV	7 (23)	0	1 (20)	2 (25)	10
Patient/public	1 (3)	0	3 (30)	5 (63)	9
Non-profit organisation (n (%))	1 (3)	0	1 (10)	0	2
Content					
General	28 (93)	3 (43)	0 (0)	6 (75)	37
Surgery	26 (87)	5 (71)	1 (10)	2 (25)	34
Needle aponeurotomy (%))	19 (63)	2 (29)	3 (30)	0 (0)	21
Collagenase injection (%))	25 (83)	2 (29)	4 (40)	2 (25)	33
Other	2 (7)	0 (0)	0 (0)	5 (63)	7
Single Focus (n (%))	23 (76)	5 (71)	8 (80)	1 (13)	37
Advertisement (n (%))	16 (53)	0 (0)	2 (20)	1 (13)	19
Video properties					
Total length (h:mm:ss)	1:57:10	0:40:24	0:27:47	0:32:17	03:37:38
Total views	152,131	105,894	147,868	47,508	453,401
Total “likes”	160	14	50	64	228

The videos deemed “misleading” discussed general disease information in 75% of cases; however, the validity of the information was frequently questionable. Videos deemed “misleading” advocated treatments with no established evidence base, compared with videos “useful to patients” (*p=*0.002). Youtube is a free video-sharing site containing over 100 million videos. Anyone can publish videos on the site regardless of their qualifications or intention. The health information available can vary from informative, to promotional, to misleading and potentially harmful. Due to the growth and popularity of the site, it could be considered a powerful tool for health education. 

Medical professionals and News/TV reports produced the videos most useful to patients; however, these were not those most viewed. Videos uploaded by patients/public that described personal experiences were viewed most often, and were most likely to contain misleading information. Therefore, viewers were more often accessing and agreeing with videos that promoted potentially misleading medical information, produced by the layperson. Videos that were “useful to patients” covered the currently accepted treatment options for DD, although few videos (27%) provided a comprehensive overview of all treatments. 

It was interesting to note a disproportionate amount of coverage on collagenase injections and needle aponeurotomies, in conjunction with the finding that 37% of these were found in videos made by medical professionals that were deemed ‘advertisements’. We speculate that the production of these videos may be influenced by the cost benefits of being able to offer a quick, ‘office-based’ alternative to surgery. Needle aponeurotomy and collagenase may not be suitable for all patients,^4^^,^^5^ and patients viewing these videos could be misled into thinking that these treatments are superior, and may be disinclined to accept surgery even if clinically indicated. 

There is accurate and useful information available on the topic of DD on Youtube, but it is interspersed with misleading and potentially harmful information; moreover, few videos provided a balanced overview of all available treatment options. Patients should be aware of the source and intent of the video, and be prepared to filter them accordingly. Patients should put preference on viewing videos uploaded by medical professionals, as we have found that they provide the most accurate information. Youtube relies on patients being able to locate quality content, and we believe there is a call for professional medical societies to produce videos that outline all available treatment modalities, including the risks and benefits of each, and to which patient group the treatments are most appropriate. So as useful patient education videos are available on YouTube but are interspersed between ones that are potentially misleading, there appears to be a disproportionate amount of information focusing on needle aponeurotomy and collagenase injections. Patients should be aware of the source and intent of the video, and put preference on viewing those produced by medical professionals. 
